# Initiation and Pathogenesis of Severe Asthma with Fungal Sensitization

**DOI:** 10.3390/cells10040913

**Published:** 2021-04-15

**Authors:** Meenakshi Tiwary, Amali E. Samarasinghe

**Affiliations:** 1 Division of Pulmonology, Allergy and Immunology, Department of Pediatrics, College of Medicine, University of Tennessee Health Science Center, Memphis, TN 38103, USA; mtiwary@uthsc.edu; 2 Children’s Foundation Research Institute, Memphis, TN 38103, USA

**Keywords:** *Aspergillus*, mouse models, mycobiome, eosinophils

## Abstract

Fungi represent one of the most diverse and abundant eukaryotes on earth, and their ubiquity and small proteolytically active products make them pervasive allergens that affect humans and other mammals. The immunologic parameters surrounding fungal allergies are still not fully elucidated despite their importance given that a large proportion of severe asthmatics are sensitized to fungal allergens. Herein, we explore fungal allergic asthma with emphasis on mouse models that recapitulate the characteristics of human disease, and the main leukocyte players in the pathogenesis of fungal allergies. The endogenous mycobiome may also contribute to fungal asthma, a phenomenon that we discuss only superficially, as much remains to be discovered.

## 1. Introduction

**Figure 1:**
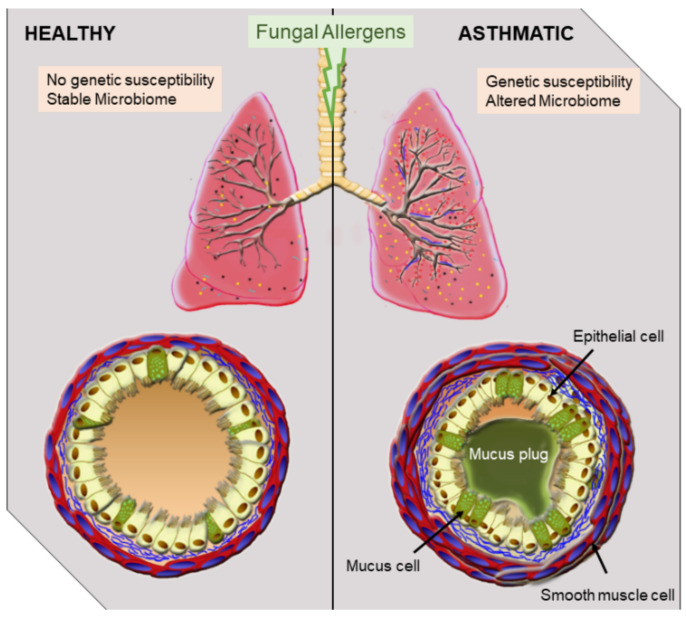
Overview of airway pathophysiology in health and asthma. Asthma triggers include fungal allergens which are abundant in the environment. While healthy individuals do not respond to inhaled fungal allergens, in the presence of confounders including genetic susceptibility and an altered microbiome as in atopic individuals, fungal exposures can lead to alterations to the mucosa. Typical occurrences after fungal exposure in asthmatics include inflammation of the airways, mucus hyperproduction, smooth muscle thickening, and remodeling events.

Symptoms of asthma have been described in ancient literature, with the first detailed description by Sir John Floyer dating back to 1698 [1]. Asthma is a respiratory syndrome with high incidence and economic burden worldwide [2,3]. This heterogenous syndrome has many subtypes and phenotypes, but typical symptoms generally include chest tightness, wheezing, and shortness of breath [4,5] that can result from narrowing of large airways due to heightened inflammation, mucus, and smooth muscle cell constriction (Figure 1). Asthma is generally classified as allergic and nonallergic, wherein allergic asthma is defined by the presence of atopy, i.e., allergen-specific IgE in plasma and positive skin-prick test to common aeroallergens [6,7,8,9]. Genetics, epigenetics, the endogenous microbiome, and environmental factors influence the development, exacerbation, and severity of allergic asthma [9,10,11]. During early development, genetics and epigenetics shape the immune system, thus playing an important foundational role in determining the allergic asthma endotype [12]. Sensitivity to indoor and outdoor aeroallergens results as a secondary trigger in individuals that are genetically prone to developing allergic asthma. Common aeroallergens include house dust mite and cockroach antigens, molds, and pollen [13].

Fungi are ubiquitous in both indoor and outdoor environments and widespread worldwide, making up the largest organic component in air particulates [14,15]. Owing to effective antifungal host mechanisms such as antimicrobial peptides, effective clearance by the mucociliary escalator, and macrophage phagocytosis, most often, fungi fail to cause infections in immunosufficient individuals. However, fungal components are important aeroallergens for asthma development, exacerbation, and severity. *Alternaria alternata*, *Aspergillus fumigatus*, and *Cladosporium herbarum* are known triggers of allergic sensitization in humans [16]. Although fungi are predominant triggers of asthma exacerbations, and severe asthma with fungal sensitization (SAFS) is often steroid resistant and difficult to treat/control, the pathophysiology of SAFS and its molecular pathways are yet to be fully elucidated. The possibility that SAFS may protect the host from subsequent respiratory virus infections [17] also elevates the need to understand the immunobiology of SAFS. We focus this review on SAFS and known effector functions of key immune players that may shape the pathogenesis of fungal allergies in the lungs as regulated by a plethora of cytokines and chemokines (Table 1).

## 2. Overview of Fungal Allergen-Mediated Immune Responses Leading to SAFS

**Figure 2:**
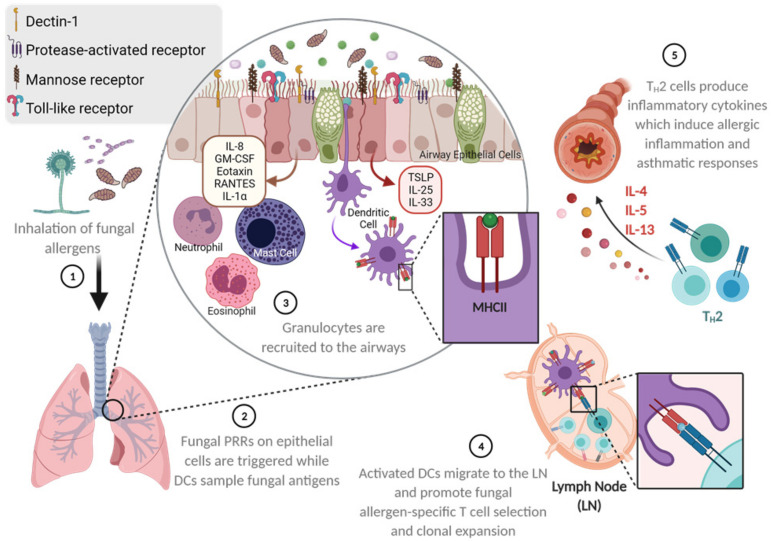
Fungal allergen-mediated early activation of the respiratory barrier as a trigger for asthma development. Environmentally ubiquitous fungi can be inhaled and travel deep into the lungs owing to their small size and surface properties. Fungal pattern recognition molecules on the respiratory epithelia may be triggered to release cytokines and chemokines that can recruit and activate a number of leukocytes. Intraepithelial dendritic cells that survey the airways may also be activated by fungal antigens and traffic into draining lymph nodes in search of antigen-specific T cells that are subsequently activated. These fungal antigen-specific T cells then accumulate at the respiratory barrier to induce resident and recruited leukocytes and structural cells to become activated and respond culminating in the characteristics of allergic asthma. Illustration drawn with BioRender.

More than 2 million species of fungi exist globally [40], but only a small fraction of these species are considered to be human pathogens [41] as the human immune system is highly efficient at safeguarding the host from environmental fungi that enter [42]. Fungus-sensitized individuals can have pathophysiologic changes in the lungs (Figure 1) resulting in the symptoms that are associated with asthma. At the respiratory interface, epithelial cells act as the primary blockade against environmental fungal colonization. Airway epithelial cells use physical barrier components such as the mucociliary escalator and antimicrobial peptides as a strategy to hinder fungal entry and germination within lung tissue [43,44]. Numerous pattern recognition receptors (PRRs) on the surface of the respiratory epithelia, including the proteinase-activated receptor (PAR), toll-like receptors (TLRs), C-type lectin receptors (CLRs), mannose receptors (MRs), and dectins, recognize a broad range of fungal antigens and are able to trigger epithelial cells to release cytokines to recruit and activate innate leukocytes to counter any invading fungi [42,45,46]. Similarly, dendritic cells (DCs) sampling the airways can become activated by fungal antigens and mucosal cytokines, allowing them to cause a fungal-specific T cell response [42,45,46] (Figure 2). Cumulatively, these immune responses in the airways can augment physiologic changes, thereby eliciting an asthma attack.

## 3. Animal Models of Severe Asthma with Fungal Sensitization

Epidemiological data have established the association of fungi with asthma [47,48,49,50,51,52]. Fungal asthma is broadly characterized by the occurrence of fungal sensitization or allergy in patients that present with asthma hallmarks [53,54]. SAFS is mainly differentiated from allergic bronchopulmonary aspergillosis by the absence of bronchiectasis and fungal growth in lungs and sensitivity to antifungal treatments [55]. The severity of SAFS can vary from mild to severe airway inflammation and airway hyperresponsiveness (AHR) [49,51,52]. SAFS is usually T_H_2 biased and characterized by inflammatory cell (predominately eosinophil) recruitment, elevated serum IgE, peribronchial and perivascular inflammation, increased AHR, mucus hypersecretion, and airway remodeling [56]. Asthmatics with fungal sensitization have similar characteristics to those that are not sensitized to fungi except for lower age of symptom onset and significantly higher levels of IgE and IL-33 in the serum [57].

Animal models that recapitulate characteristics of human asthma are important, as they provide opportunities to dissect the underlying mechanisms of asthma pathology. Ovalbumin (OVA)-induced asthma-like inflammation, the first mouse model for asthma, is more than 100 years old, and replicates a number of features of allergic asthma. However, there are several arguments against this model due to two main shortcomings. Firstly, OVA is not a clinically relevant aeroallergen; secondly, chronic exposure to OVA may lead to immune tolerance and less robust inflammation [58,59]. However, OVA is still a commonly used antigen to induce acute airway inflammation, largely due to the ease of use and the plethora of research reagents (including mouse strains) for immune assays that have been developed using OVA antigen. In the past two decades, however, more clinically relevant antigens, including fungal antigens, have been used effectively to model the characteristics of allergic asthma in mice [60] owing to the ubiquity and clinical relevance of fungi as aeroallergens. Approximately 80% of asthmatics in the United States show positive skin tests for one or more fungal allergens [61,62]. Compared with grass and pollen, fungal conidia have 1000-fold higher exposure and are among the most important clinically relevant allergens for asthma [55,62].

The establishment of a mouse model with a clinically relevant allergen, *Aspergillus fumigatus*, occurred in 1984. This model uses extract from cultured *A. fumigatus* to develop the immune response during allergen exposure. Furthermore, researchers developed conidia-based models to recapitulate the pathophysiology of fungal asthma. Various routes of conidia delivery of different fungi, including intranasal (IN), intratracheal (IT), or inhalation (IH) challenge, have been attempted [26,35,63,64]. Havaux et al. performed IN challenge of BALB/c mice with resuspended *A. alternata* and *C. herbarum* spores after sensitization. The model showed immune cell infiltration into the airway, perivascular and peribronchial eosinophilic inflammation, and increased AHR and goblet cell metaplasia [64]. As exposure to this airborne fungus nearly doubles the odds of experiencing asthma symptoms [65] *Alternaria* mouse models are important. *Alternaria* is independently capable of inducing allergic inflammation in mice and also enhances the strength and T_H_2-polarization in mice inoculated with other allergens [66,67].

Fungal exposure in humans occurs through the inhalation of airborne dry fungal particles. As fungi are complex, with several stages in their life cycle in which physical changes during growth and germination result in variations in the antigenic signatures [68], the ability to mimic the natural nature of inhaled fungi is important to model SAFS. To date, *A. fumigatus* is the only clinically relevant fungus utilized in dry inhalation models. Hoselton et al. developed an IH fungal asthma model by exposing fungal extract-sensitized BALB/c mice to dry unmanipulated airborne *A. fumigatus* conidia for 10 min. This exposure strategy led to AHR, mixed granulocytic airways inflammation, goblet cell metaplasia, increased serum IgE, reversible airway wall fibrosis, and smooth muscle hyperplasia [35]. A second IH exposure administered two weeks following the first led to marked eosinophilia and worsening of the above characteristics in C57BL/6 mice [69], a strain that has a T_H_1 immune bias and therefore difficult to model AHR in [70,71]. Samarasinghe et al. compared the asthma output in the IH and IT models of fungal asthma in C57BL/6 mice and showed that IH challenge leads to more robust eosinophilic inflammation, serum IgE, and airway wall remodeling events compared to IT challenge with suspended conidia [63]. However, sensitization to whole fungal extract is required for a robust and long-lasting asthma phenotype in *Aspergillus* models; the use of live conidia for IH elicits a robust immune response compared to irradiated (dead) conidia [72]. Buskirk et al. exposed BALB/c mice to a specific amount of *A. fumigatus* conidia with an acoustical generator, and showed that inflammation and goblet cell metaplasia peaked two days after the final exposure [73]. In summary, IH exposure to *A. fumigatus* recapitulates the hallmarks of SAFS observed in patients.

## 4. Mechanisms of SAFS Induction at the Respiratory Barrier

**Figure 3:**
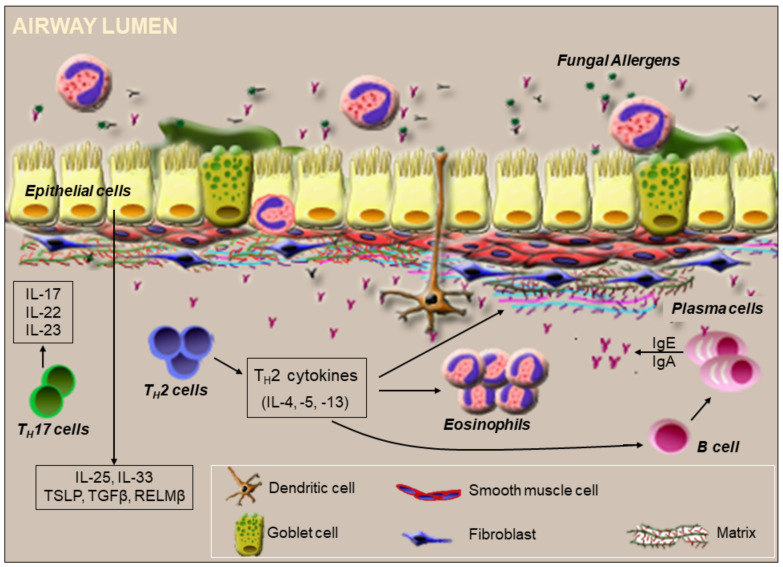
Immunologic events orchestrated by fungal exposure at the airway surface. Fungal conidia and products bind to receptors present on epithelial cells. Activated epithelial cells release cytokines and growth factors that are responsible for T_H_2 cell recruitment. Fungal proteases may also disrupt the tight junctions of the epithelial barrier, thus inducing membrane permeability. Newly differentiated T_H_2 or T_H_17 cells induced by dendritic cells activated at the barrier arrive at the respiratory barrier to regulate local immune responses to the fungal exposure. T_H_2 cells promote differentiation of B cells into plasma cells, which secrete IgE in the presence of IL-4 and IL-13, while IL-5 supports the survival of recruited eosinophils. T_H_2 cytokines also induce airway remodeling by altering the extracellular matrix. Conversion of hyaluronan from high to low molecular weight forms can further promote B cell recruitment and activation to secrete neutralizing antibodies that also activate leukocytes like mast cells at the respiratory barrier. IL-17 and IL-22 produced by T_H_17 cells also enhance inflammation. On the luminal end, mucus and eosinophil extracellular nets may cause fungal entrapment while eosinophil degranulation may neutralize fungal antigens.

Major asthma-causing fungal allergens belong to genera *Alternaria* and *Aspergillus* [55,74]. *Aspergillus* conidia are roughly <3 μm [75], i.e., small enough to penetrate the deep bronchoalveolar spaces in the lower airway. Fungal conidia can interact with the airway epithelial barrier, triggering inflammatory signals in response. The major pathogen associated molecular patterns (PAMPs) of fungi include chitin, β-glucans, proteases, glycosidases, and fungal nucleic acids. Receptors for fungal products include TLRs, CLRs, PARs, and receptor for glycation end products (RAGE). Figure 3 illustrates some inflammatory cascades that can be initiated during fungal interaction at the lung epithelial cell (LEC) interface.

Fungal components such as β-glucan can be recognized by PRRs on DCs independently or in conjunction [76,77], and fungal-antigen activated DCs can go on to trigger both T_H_2 and T_H_17 cells [55,78]. Fungal β-glucans also induce dectin-1-dependent IL-6 production in murine LECs [79]. Conidia surface constituent chitin induces TLR-2-dependent IL-17A production by murine macrophages in vitro [80]. Moreover, exposure of murine LECs to fungal chitin induces production of CCL2 [81,82]. LECs also produce IL-25, IL-33, and thymic stromal lymphopoietin (TSLP) in response to fungal allergens [45,46], which regulate downstream immune responses, as the deletion of one or more of these cytokine genes in mice leads to reduced inflammation in response to chitin [83]. Eosinophils, mast cells, and T-cells also secrete IL-25 [84], which is important to perpetuate airway eosinophilia [85], while IL-33 plays a significant role in eosinophilia, goblet cell hyperplasia, and airway remodeling in SAFS [86]. Fungal allergens induce IL-33 secretion in LECs through oxidative stress responses and NADPH oxidase DUOX1 mediated activation of calpain-2 and EGFR signaling [87]. IL-33 promotes CD11b, β-glucan, and ICAM-1 expression on eosinophils [88], which are important for eosinophil activation, and also promote eosinophil survival [89]. While the presence of T_H_2 and T_H_17 cytokines in the airways is prominent, early pro-inflammatory cytokines such as IL-1 and TNF-α are also produced in response to fungal sensitization and challenge [90,91]. Resistin-like molecule (RELM)-β is a secreted protein that is abundant in the gut, but is also produced by LECs [92,93]. Architectural changes in the lungs including goblet cell metaplasia and peribronchial fibrosis increase in the absence of RELM-β in *A. fumigatus* allergen-sensitized and challenged mice [94]. Therefore, LEC products may also impede immunopathologic changes triggered by fungal allergens and therefore be beneficial to mucosal immunity.

Fungal spores and conidia produce a variety of proteases during their life cycle, and secreted proteases are often immunogenic. These proteases are recognized by PARs [95] and can be pro-inflammatory and lead to tight junction disruption between LECs [53,96,97,98], thereby compromising the physical barrier. The deletion of lung tight junction protein claudin-18 in mice causes elevated serum IgE levels and increased AHR after *Aspergillus* sensitization [98]. Fungal components also induce ion secretion by stimulating the CFTR and Ca^2+^ channels on epithelial cells thereby affecting mucociliary clearance in the airways [99]. Due to protease-induced irritation, LECs in injured lungs release IL-33, which interacts with its receptor (ST2) on recruited leukocytes including type 2 innate lymphoid cells, resulting in further enhancement of the T_H_2-type cytokine production and immune responses [100,101]. A T_H_2-biased immune response and airway remodeling can be triggered by IN delivery of *A. fumigatus* matrix metalloprotease Asp f 5 and serine protease Asp f 13 [102]. Typically, fungus-epithelial barrier interaction activates LECs to relay a T_H_2 bias, which includes a specific type of cytokine/chemokine response and immune cell profile.

## 5. T Cell Response to Fungal Allergen Exposure in the Airways

Immune responses that occur in the lungs in response to fungal allergens involve mast cells, basophils, eosinophils, innate lymphoid cells (ILCs), M2-polarized macrophages, and T_H_2 cells, all of which can produce T_H_2-type cytokines like IL-4, IL-5, and IL-13 [103,104,105,106,107]. Inhaled fungal allergens can be endocytosed by DCs, which process and present fungal antigens using major histocompatibility complex II (MHC II). Activated DCs can then migrate to draining lymphoid organs, where they control the differentiation of CD4^+^ T cells into T_H_2 cells [107]. The differentiation of CD4^+^ T cells into T_H_2 cells depends upon TSLP, CCL17, and CCL22. Once activated, T_H_2 cells produce inflammatory cytokines IL-4, IL-5, IL-9, and IL-13 (Figure 2). T_H_2 cells also interact with allergen-specific B-cells, and IL-4 and IL-13 produced by T_H_2 cells cause B cell class switching to IgE production [108,109]. Major T_H_2-type cytokine, IL-13, induces goblet cell metaplasia, fibrosis, and AHR [110,111], and, in conjunction with IL-5, promotes the proliferation and survival of eosinophils in the airway [112]. In addition to these cytokines, chemokines CCL5 and CCL11 stimulate eosinophil recruitment into the airway [108]. Cumulatively, these immune responses culminate pathophysiologically as airway constriction, a major hallmark of asthma symptoms (Figure 2).

Recent research implicates IL-17 production by T_H_17 cells in SAFS (Figure 3). In addition to T_H_17 cells, ILCs, B-cells, neutrophils, γδ T cells, and natural killer T cells secrete IL-17 [113,114,115,116]. IL-17 orchestrates asthma pathophysiology including inflammation (primarily neutrophil recruitment), smooth muscle proliferation, and fibrosis. These functions of IL-17 are regulated through the induction of type 2 cytokines, proallergic chemokines, and proinflammatory cytokines [117]. Regulatory T cells are activated by TLR-2 signaling in response to fungal antigens [118], and Tregs can elevate the functions of T_H_17 cells or help suppress the functions of T_H_2 cells depending on the level of fungal antigens at the mucosa [119]. Intriguingly, TLR-6 upregulated during *A. fumigatus* exposure [120] contributes to IL-17A and IL-23 production by T_H_17 cells in response to fungal allergens during asthma [32] (Figure 3).

## 6. Eosinophils in SAFS

Eosinophils are granulocytes with distinct, acentric bilobed nuclei and cytoplasmic granules with cytotoxic properties. Since its first description by Paul Ehrlich in 1879, eosinophils have been shown to have immunoregulatory and homeostatic functions [121]. During SAFS, T_H_2 cytokines IL-4, IL-5, and IL-13, as well as fungal antigens, stimulate the production of eosinophil chemoattractant, CCL11 by LECs [122] (Figure 2). The most potent growth factor and chemoattractant for eosinophils is IL-5, which is sensed by the IL-5R complex expressed on both eosinophils and basophils [123,124]. Therefore, IL-5 produced by T_H_2 cells induces differentiation, proliferation, and maturation of eosinophils during fungal allergies. As eosinophilia is a common manifestation in SAFS patients, biologics that inhibit the effects of IL-5 (mepolizumab, reslizumab, and benralizumab) may be efficacious at alleviating asthma symptoms [125]. Once recruited, eosinophils may perform several functions in situ in response to fungal antigens, as they have been shown to directly bind *Alternaria* [126], release granule proteins in response [127], and kill *A. fumigatus* [128]. Eosinophils also produce extracellular DNA traps in response to *A. fumigatus*, which are not fungicidal [39], but may be immunoregulatory in the context of the allergic airways.

Eosinophils may contribute to fungal asthma pathophysiology by increasing AHR, activating T_H_2 cells, and inducing airway remodeling. Eosinophils induce AHR by releasing cytokines such as IL-13 [129] and inducing mast cell and basophil degranulation [130,131]. Intriguingly, β-integrin CD11b on the surface of human eosinophils specifically binds to fungal wall β-glucan, causing eosinophil activation and degranulation which may promote fungal killing but could potentially contribute to asthma pathophysiology [126]. Eosinophils induce airway remodeling by releasing profibrotic mediators such as TGF-β in the presence of IL-4 [132]. TGF-β induces extracellular protein production, fibroblast proliferation, and smooth muscle cell proliferation [133,134]. However, eosinophil depletion by anti-IL-5 treatment does not completely suspend airway remodeling [135,136], as a number of cell types in the lungs, such as LECs and macrophages, produce TGF-β, thereby limiting our understanding of the exact role of TGF-β produced by eosinophils during fungal asthma. As RELM-β is important in lung fibrosis in response to *A. fumigatus* [94], and LECs [137] and to a lesser degree leukocytes (including eosinophils) [138] produce RELM-β, it is highly likely that additional mechanisms may be activated to induce subepithelial fibrosis in response to fungi. Eosinophils are also a significant source of IL-17 and IL-23 after fungal exposure [38]. As one of the most highly recruited leukocytes in fungal asthma [139], eosinophils can indirectly contribute to asthma pathophysiology through crosstalk with T_H_2 cells and other leukocytes in situ by releasing a plethora of cytokines [140].

According to the current paradigm, peripheral eosinophils are considered end-stage effector cells that play an active role in the initiation and prolongation of allergic asthma pathology. Additionally, many organs in healthy individuals have resident eosinophils [141]. This evidence questions the deleterious end-stage effector role of eosinophils. A large number of studies have demonstrated the tissue repair/remodeling, tissue homeostasis, and developmental functions of eosinophils [141], and their role in regulating antiviral defense mechanisms during fungal asthma [91,142,143]. Due to the multiple beneficial roles of eosinophils, Lee et al. proposed the new hypothesis “local immunity and/or remodeling/repair in both health and diseases” (LIAR), wherein eosinophil recruitment during both healthy and pathological conditions, like fungal asthma, occurs to maintain tissue homeostasis [121]. More recently, eosinophils have been shown to play host protective functions against respiratory pathogens [144] that further support a role for eosinophils as an ally to respiratory health. As significant eosinophil reduction through biologics does not always result in the total ablation of asthma pathophysiology in patients [145], it may be necessary to consider the impact of long-term eosinophil depletion on human health.

## 7. B Cells in SAFS

The discovery of B cells occurred in the mid 1960s. B cells significantly increase in the blood stream and bronchial mucosa of asthmatic patients [146,147,148]. Mice rendered allergic to *A. fumigatus* also show markedly increased B cells in the airways [103,117] and mediastinal lymph nodes [37]. T_H_2 cytokines induce antibody class switching from IgG to IgE [108,109]. IgE produced by B cells in fungal asthma bind to Fcε receptors on mast cells and cross link upon contact with allergenic antigens, causing mast cell degranulation and release of histamine and prostaglandins [149,150]. In our *A. fumigatus*-based murine fungal asthma model, B cells produce high amounts of IgA and IgE, which are localized to peribronchial and perivascular spaces in the lungs [37]. This finding indicates local production of antibodies by mucosal B cells during fungal asthma. Ghosh et al. determined that B cells play a significant role in regulating inflammation during fungal asthma, as mice deficient in mature B cells (J_H_^−/−^ strain) have elevated pro-inflammatory cytokines (IL-6 and IL-17A) and reduced canonical T_H_2 cytokines [117]. Using a combination of allergens including OVA, house dust mite, *Aspergillus* and *Alternaria*, Drake et al. showed that J_H_^−/−^ mice had reduced lung eosinophilia, increased AHR, and elevated cytokines and chemokines compared to their wild-type counterparts [104,151]. Fungal allergic inflammation promotes the enzymatic cleavage of hyaluronan to its low molecular mass form which attracts B cells through CD44 engagement [103]. Together, these findings highlight the possibility that B cells may play an immunomodulatory role during fungal allergic asthma that surpasses that of antibody production.

## 8. Commensals and Allergic Asthma

The human body harbors a vast amount of diverse microbial communities. The complex interactions between the microbiome and host in the gastrointestinal tract, skin, and respiratory system is pivotal in development and health [152,153,154,155]. Historically, the lungs were considered sterile in healthy individuals. However, recent research revealed that the lungs of healthy individuals harbor low levels of diverse microbiota, mainly comprised of bacteria. To date, data on the fungal population or mycobiome of the lungs are scarce. Charlson et al. performed a comparative study on bronchoalveolar lavage samples from healthy individuals and lung transplant recipients, and found that healthy individuals showed minimal fungal ITS amplification compared to lung transplant recipients, surmising that antibiotics and immunosuppressants prescribed to lung transplant recipients likely caused the increased abundance of *Candida* spp., *Aspergillus* spp. and *Cryptococcus* spp. [155]. An individual’s microbiome contributes to immune system development, while dysbiosis in the lung may contribute to the etiology of allergic diseases like asthma [156,157]. The unique fungal population in the airway is also associated with an increased risk of allergic asthma. In a human cohort study, the sputum of asthmatic patients showed 90 common fungal species including *Psathyrella candolleana, Malassezia pachydermatis, Termitomyces clypeatus* and *Grifola sordulenta*, whereas the sputum of control subjects showed 46 common species [57,158]. However, sputum samples do not truly represent the lung microbiome because of possible contamination from gut and oral microbiomes. Therefore, the lung mycobiome with respect to allergic asthma requires further exploration.

Due to the gut–lung axis, gut microbiota can have a peripheral impact on the development and regulation of the lung immune system. For example, dysbiosis in the gut may increase the risk of allergic diseases such as asthma [152]. At present, most reports, including ours [159], have characterized the bacterial microbiome in the context of allergic asthma, and very few have characterized the mycobiome. In a recent mouse study, dysbiosis in the gut mycobiome induced by fluconazole, an antifungal drug, reduced the number of *Candida* spp. and expanded commensal fungi *Aspergillus* spp., *Wallemia* spp., and *Epicoccum* spp. These antifungal-treated mice demonstrated severe allergic asthma in response to intratracheally delivered house dust mite antigen with elevated eosinophil infiltration, serum IgE, and cytokines IL-4, IL-5 and IL-10 [152]. Intriguingly, oral supplementation with commensal fungus *Wallemia mellicola* was sufficient to recapitulate characteristics of allergic asthma in mice [160]. These studies confirm that the gut mycobiome plays an important role in peripheral immune responses including those in the lungs. The contribution of the mycobiome may help protect the barrier from invading environmental fungi and other infectious agents. Mannan derived from *Saccharomyces cerevisiae*, a component of the human gut mycobiome [161], can promote airway epithelial cell spreading and wound healing [162]. However, the use of mannan therapeutically did not reduce the pathogenesis of *A. fumigatus*-induced fungal asthma [163]. Our understanding is limited regarding the complex interplay between the gut mycobiome and development/regulation of the peripheral immune system during fungal asthma.

The reduction in parasitic infections has been considered to, at least partially, contribute to the increase in allergic diseases in the Western world. While both positive and negative impacts of parasites on the development of allergies have been demonstrated [164,165], information that is specific to the relationship between parasitic infections and the development of fungal allergies is limited. Mice pre-infected with gut parasite *Heligmosomoides polygyrus* had altered responses to *A. fumigatus* sensitization and challenge based on the age of parasite infection [166], suggesting that the age-related maturity of the immune system has a direct impact on the development of fungal allergies during an active parasite infection. As gut parasites alter the gut microbiota which, in turn, affect the pathogenesis of allergic asthma [167], the interrelationship between parasites and the development of fungal asthma may be multifaceted and remain to be fully elucidated.

## 9. Conclusions

Fungal allergies are a growing concern, as fungi are found both indoors and outdoors in abundance in rural and city environments, making avoidance strategies difficult. Despite the millions of fungal species found in the global environment, only a small fraction is known cause human infections. Even fewer species are known allergens even with the high incidence of fungal allergies in atopic individuals [168]. Why some patients are susceptible to fungal sensitization while others are not remains to be determined. As fine classifications of immune responses at the airway barrier, and differentiation of immune responses at the initiation and perpetuation of fungal sensitization are not well understood in humans, animal models that can recapitulate the hallmarks of SAFS in humans are of immense importance. Elucidating the correlation between environmental fungal load and sensitization may also be beneficial to determine if immune tolerance may be induced in the clinical setting to offset sensitization.

Most SAFS patients have heightened eosinophilic inflammation [137]. While SAFS is generally nonresponsive to corticosteroids, the advent of anti-eosinophilic biologics has led to better management of asthma severity in SAFS patients [169]. The knowledge that eosinophils play a host-protective role during virus infections [142,170], including IAV infections [91,141,171] and possibly SARS-CoV-2 infection [172], raises the question of whether fungal allergen-induced eosinophilia has some benefit, at least seasonally, and if so, what the possible long-term complications may be of eliminating eosinophils in SAFS patients [173]. Much remains to be discovered on the immune pathogenesis of fungal allergies and the consequences of fungal allergic disease on immune responses to subsequent or concomitant infectious stimuli.

## Figures and Tables

**Table 1:** Immunological and Inflammatory Events Following Fungal Exposure.

Source	Mediator	Effect	References
Fungi	Serine proteases	Membrane permeability Disruption of tight junctions Airway smooth muscle constriction	[18,19,20,21,22,23,24]
Epithelial cells	Interleukins -25 and -33 TSLP TGF-β	Inflammation Leukocyte activation Airway remodeling	[25,26,27,28,29,30]
Dendritic cells	Pattern recognition receptors Interleukin-6 TNF-α	Fungal recognition Interleukin-17A production Neutrophil recruitment	[31,32,33,34]
T_H_2 cells	Interleukin-4 Interleukin-5 Interleukin-13	Inflammation B cell class switching Eosinophil activation Mucus cell activation	[26,27,31,35]
T_H_17 cells	Interleukin-17A	Neutrophil recruitment Epithelial cell activation	[33]
Plasma cells	Immunoglobulin E Immunoglobulin A	Mast cell activation Fungal neutralization	[26,27,31,35,36,37]
Eosinophils	Interleukins -17 and -23 DNA traps	Inflammation Fungal neutralization	[38,39]

## Data Availability

Not applicable.
